# 5‐Thiohistidine *N*‐Acetyltransferase from *Proteiniphilum Saccharofermentans*

**DOI:** 10.1002/cbic.202400439

**Published:** 2025-10-07

**Authors:** Cangsong Liao, David Lim, Gladwin Suryatin Alim, Florian P. Seebeck

**Affiliations:** ^1^ Department of Chemistry University of Basel Mattenstrasse 22 Basel 4002 Switzerland; ^2^ Molecular Systems Engineering National Competence Center in Research (NCCR) Basel 4058 Switzerland

**Keywords:** acetyltransferase, marine natural products, ovothiol

## Abstract

Ovothiol A is a 5‐thiohistidine derivative biosynthesized by a broad range of prokaryotic and eukaryotic organisms. Its redox‐active mercaptoimidazole side chain is believed to protect cells from oxidative stress. The three enzymes that produce ovothiol A from histidine, cysteine, and *S*‐adenosyl methionine have been identified and characterized. In contrast, no enzymes are known that produce other 5‐thiohistidine derivatives. Here, a small family of acetyl‐coenzyme A‐dependent transferases is described that produce *N*‐acetyl‐5‐thiohistidine. The discovery of these enzymes from *Proteiniphilum saccharofermentans* and related Bacteroidota provides evidence that the 5‐thiohistidine class may be structurally and functionally more diverse than previously thought.

## Graphical abstract

*Proteiniphilum saccharofermentans*, a Gram‐negative facultative anaerobe, produces an *N*‐acetyl transferase (5THNAT) that acetylates 5‐thiohistidine using acetyl‐CoA. This reaction competes with OvoC‐mediated *N*π‐methylation of the same substrate. The production ratio of ovothiol to *N*α‐acetyl 5‐thiohistidine is likely regulated by the relative availability of SAM and acetyl‐CoA.

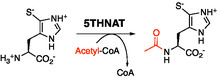

## 1 Introduction

**Figure 1:**
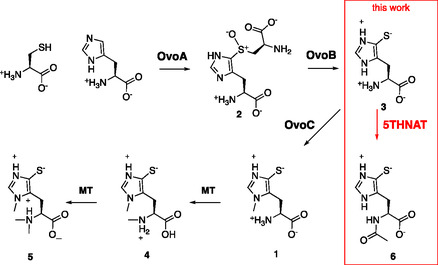
Biosynthesis of Ovothiol A, B, and C (**1**, **4**, **6**) by a sulfoxide synthase (OvoA), a PLP‐dependent broad specificity β‐lyase (OvoB), and SAM‐dependent methyltransferases (OvoA and unknown MT). The *N*‐acetyl transferase from *P. saccharofermentans* (5THNAT) catalyzes *N*α‐acetylation of 5‐thiohistidine.

Ovothiol A (**1**) is a sulfur‐containing natural product that has been isolated from marine invertebrates,^[^ ^1^ ^]^ ameba,^[^ ^2^ ^]^ and trypanosomatids.^[^ ^3^ ^]^ These organisms produce ovothiol from histidine, cysteine, and *S*‐adenosyl methionine (SAM) in three enzyme‐catalyzed steps (**Figure** **1**).^[^ ^4^ ^]^ The N‐terminal module of the ovothiol synthase OvoA is a sulfoxide synthase (EC 1.14.99.52) that catalyzes oxidative coupling between the side chains of histidine and cysteine to form a sulfoxide intermediate (**2**).^[^ ^5^ ^]^ A broad specificity β‐lyase (OvoB, EC 4.4.1.‐) eliminates the cysteinyl moiety to produce 5‐thiohistidine (**3**).^[^ ^6^ ^]^ Finally, the C‐terminal SAM‐dependent methyltransferase domain of OvoA—also referred to as OvoC—methylates **3** at the Nπ of the imidazole ring (EC 2.1.1.‐).^[^ ^6^ ^]^ The crystal structures of these three enzymes have recently been described.^[^ ^6^, ^7^ ^]^

A wide variety of marine and terrestrial species growing in aerobic or anaerobic environments, including bacteria, fungi, algae, and animals, encode OvoA homologs,[^1^, ^5^, ^8^] suggesting that ovothiol either serves a fundamental function that is important to many different life forms or may assume several different functions. Yet, little is known about the specific processes that make ovothiol a useful, if not essential, metabolite. The redox‐active mercaptoimidazole side chain of ovothiol is suggestive of an antioxidant role.^[1a]^ Ovothiol and its selenium‐containing derivative, ovoselenol,^[^ ^9^ ^]^ may protect cells by attacking electrophilic oxygen species such as singlet oxygen or hydrogen peroxide in polar reactions,^[^ ^10^ ^]^ or by engaging in one‐electron exchanges with harmful radicals.^[^ ^11^ ^]^ Despite these possibilities, there is little convincing evidence that these nonspecific small‐molecule reactions are relevant or essential under physiological conditions.^[^ ^12^ ^]^

Relevant functions of ovothiol may depend on interactions with macromolecules. For example, early discoveries indicate that ovothiol A may regulate photosynthetic activity in the halophile green micro‐alga *Dunaliella salina* by interacting with the chloroplast coupling factor 1.^[^ ^13^ ^]^ However, little is known about proteins that specifically sense, transform, or depend on 5‐thiohistidines, despite evidence that such enzymes should exist. Certain marine species, including diatoms, sea urchins, and starfish, contain ovothiol B (**4**), and ovothiol C (**5**),^[^ ^14^ ^]^ indicating that these organisms—or possibly some of their microbial symbionts—likely contain methyltransferases that methylate the α‐amino group of ovothiol A. Similarly, the documentation of complex 5‐thiohistidine‐containing marine secondary metabolites may be evidence of enzyme‐catalyzed thiohistidine conjugation.[^1^, ^15^]

The discovery of 5‐thiohistidine‐utilizing enzymes is a critical prerequisite to understanding the biological functions of 5‐thiohistidines beyond what may be gleaned from their inherent reactivity. As a step in this direction, we describe in this report an acetyl‐coenzyme A (CoA)‐dependent *N*‐acetyl transferase from the gram‐negative bacterium *P. saccharofermentans* that attaches an acetyl group to the α‐amino function of **3** to produce *N*α‐acetyl 5‐thiohiohistidine (**6**). Because of this activity, we term this novel enzyme 5‐thiohistidine *N*‐acetyltransferase (5THNAT).

## 2 Results and Discussion

**Figure 2:**
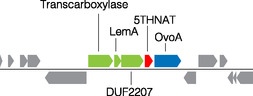
Genome neighborhood of 5THNAT from *P. saccharofermentans*.^[^ ^5^ ^]^

To identify new enzymes that may be involved in modifying or utilizing 5‐thiohistidines, we inspected the genome neighborhood of bacterial genes that code for homologs of OvoA from *Erwinia tasmaniensis*.^[^ ^5^ ^]^ Using the Genome Neighborhood Tool,^[^ ^5^ ^]^ we identified the OvoA homolog (A0A1R3SZC8) from *P. saccharofermentans* encoded together with a LemA family protein (A0A1R3T6Q4), a DUF2207 protein with an N‐terminal leader sequence (A0A1R3T4M7), a putative transcarboxylase (A0A1R3SVB5),^[^ ^16^ ^]^ and a putative acetyltransferase which we later identified as 5THNAT (A0A1R3T2J6, **Figure** **2**). LemA proteins may be involved in cell‐membrane restructuring.^[^ ^16^ ^]^ A DUF2207 protein from *Streptococcus mutans* has been implicated in stress tolerance and biofilm formation,^[^ ^17^ ^]^ and transcarboxylases are biotin‐dependent enzymes that transfer CO_2_, for example, from methylmalonyl‐CoA to pyruvate to form oxaloacetate and propionyl‐CoA.^[^ ^16^ ^]^ This five‐gene cluster is not a conserved motif (Figure S1, Supporting Information). Our Genome Neighborhood analysis^[^ ^5^ ^]^ revealed only four genomes with a transcarboxylase and OvoA encoded in the same operon. Furthermore, even highly related pairs of DUF2207 and LemA proteins (E‐Value < 8e‐148) occur in organisms, for example *Pseudalgibacter alginicilyticus*, that do not encode any OvoA homolog. Hence, we conclude that the activities of the DUF2207, LemA, and the transcarboxylase in *P. saccharofermentans* are not directly linked to 5‐thiohistidine biochemistry.

In contrast, we identified over 50 genomes with OvoA homologs co‐encoded with an acetyltransferase that shows limited but significant similarity (<30% sequence identity) to members of the GCN5‐related N‐acetyltransferase family, which catalyzes acetyl transfer from acetyl‐CoA to *N*‐nucleophiles.^[^ ^18^ ^]^ Although the best‐documented representatives of this superfamily are protein acetyltransferases,^[^ ^19^ ^]^ numerous transferases that acetylate small molecules have been identified and crystallized, including *N*‐acetyltransferases of canavanine (8OSP),^[^ ^20^ ^]^ nourseothricin (5C82)^[^ ^21^ ^]^ phosphinothricin (5T7E),^[^ ^22^ ^]^ polyamines (8A9O, 4R9M),^[^ ^23^ ^]^ and desacetylmycothiol (2C27).^[^ ^24^ ^]^

**Figure 3:**
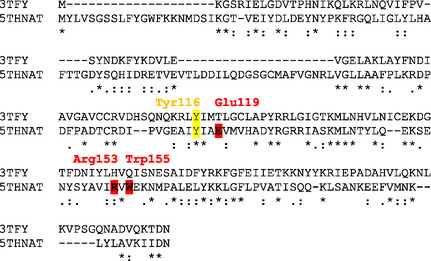
Sequence alignment of human N‐terminal acetyl‐transferase (3TFY) and the 5‐thiohistidine N‐acetyl transferase from *P. saccharofermentans* (SCD20259.1). The CoA‐binding residues (green) and a catalytic base are conserved (yellow); central residues in the acetyl‐acceptor binding pocket are different (red). 5THNAT residues are numbered according to the Uniprot entry A0A1R3T2J6.

Among the *N*‐acetyltransferases with published structures, the human amino‐terminal acetyltransferase in complex with CoA and a peptide substrate shares most similarities with the homolog from *P. saccharofermentans* (3TFY, seq. Id: 23%).^[^ ^25^ ^]^ Comparison of this crystal structure with the α‐fold structure of 5THNAT (A0A1R3T2J6) revealed that most residues interacting with CoA are conserved. In addition, an active site Tyr in the human enzyme (Tyr73), which has been identified as the catalytic base that deprotonates the Nα‐amino function of the substrate, is conserved in 5THNAT (Tyr116, yellow, **Figure** **3**).^[^ ^25^ ^]^ Key differences appear in the binding pocket for the acetyl acceptor substrate. Glu119, Arg153, and Trp155 (red) are strictly conserved among OvoA‐associated acetyltransferases, suggesting that these residues are important for recognizing their common acceptor‐substrate. Taken together, the structural similarities with known *N*‐acetyl transferases and the genomic association with OvoA support the idea that 5THNAT belongs to a small family of CoA‐dependent *N*‐acetyl transferases with a function that is related to 5‐thiohistidine or ovothiol.

**Scheme 1:**
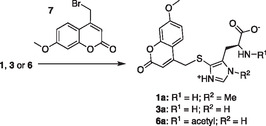
Labeling thiohistidine derivatives with Bromomethyl‐7‐methoxycoumarin (7) to simplify detection and quantification by HPLC.

**Figure 4:**
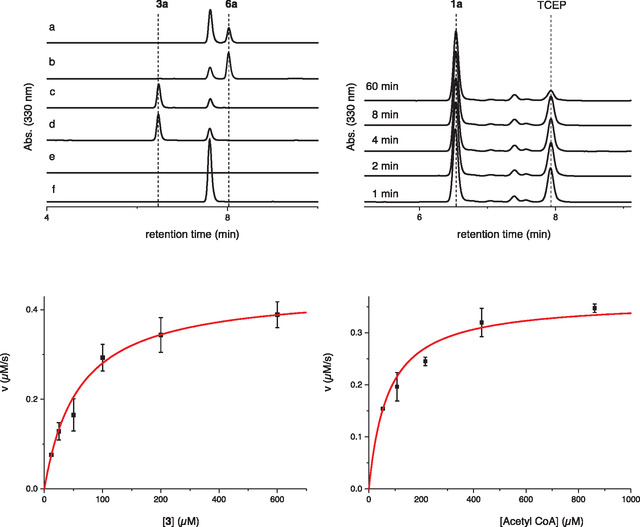
In vitro activity of 5THNAT. Top left: Solutions were incubated for 60 min at 25 °C and analyzed by HPLC after alkylation by **7**. These solutions contained 50 mM phosphate buffer pH 8.0, 20 mM NaCl, 2 mM TCEP and a) authentic **6** synthesized following a published protocol;^[^ ^30^ ^]^ b) 1 mM acetyl‐CoA, 500 μM **3** and 10 μM enzyme; c) same as (b) but without enzyme; d) authentic **3**, e) 1 mM acetyl‐CoA; f) no addition. Top right: Aliquots were taken from a similar reaction containing **1** instead of **3** after 1, 2, 4, 8, and 60 min. No consumption of **1** was detected under these conditions. Consumption of TCEP is likely caused by the rapid autooxidation of **1** in the presence of O_2_ and traces of Fe^II^ or Cu^I^. Bottom: Determination of the kinetic parameters for 5THNAT‐catalyzed acetylation of **3** using acetyl‐CoA as a co‐substrate.

To examine the nature of this relationship, we produced 5THNAT from *P. saccharofermentans* in *Escherichia coli*. The recombinant protein was purified via nickel nitrilotriacetic acid agarose affinity chromatography and analyzed by sodium dodecyl sulfate‐polyacrylamide Gel Electrophoresis (Figure S2, Supporting Information). We determined the activity of this enzyme by high‐performance liquid chromatography (HPLC), using UV‐based quantification of coumarin‐derivatized 5‐thiohistidines following a published protocol.^[^ ^26^ ^]^ Briefly, 100 μl reactions containing 50 mM phosphate buffer (pH 8.0), 20 mM NaCl, 2 mM TCEP,1 mM acetyl‐CoA, 500 μM **3** or **1** and 10 μM enzyme were incubated at 26 °C. After 1, 2, 4, 8, and 60 min, we quenched 10 μl reaction aliquots by mixing with 40 μl of 1:3 Dimethyl sulfoxide and acetonitrile solution containing 5 mM 4‐bromomethyl‐7‐methoxycoumarin (**7**, **Scheme** **1**). After 30 min, these aliquots were acidified by adding 50 μl of 0.1% TFA in H_2_O. The concentration of methyl‐7‐methoxycoumarin‐modified derivatives of **3** (**3a**), **6** (**6a**), and **1** (**1a**) in these samples was determined by HPLC analysis at 330 nm (**Figure** **4**).

This analysis revealed that 5THNAT acetylates **3** to form **6**, but does not modify **1**, suggesting that 5THNAT‐catalyzed *N*α‐acetylation and OvoA‐catalyzed *N*π‐methylation are competing rather than complementary fates of **3,** the relative efficiencies of which may be regulated by cellular SAM/SAH ratio and the availability of acetyl‐CoA. Consistent with this complementarity, some 5THNATs are co‐encoded with truncated OvoA homologs that miss the C‐terminal methyltransferase module, suggesting that these organisms only depend on the function of **6** but not **1** (Table S1, Supporting Information).

Using a similar HPLC‐based assay, we confirmed that histidine and 2‐thiohistidine are not acetylated by 5THNA under comparable reaction conditions, even after 20 h (Figure S3, Supporting Information). When we incubated the sulfoxide intermediate **2** with 10 μM 5THNA and 1 mM acetyl‐CoA for 18 h, we detected a new product (Figure S4 and S5, Supporting Information). However, this product formed at least 100‐fold more slowly than **3**, indicating that **2** is not the preferred substrate.

To quantify the *N*‐acetyltransferase activity, we determined the Michaelis‐Menten parameters of 5THNA. First, we measured the rate of product formation by 0.2 μM enzyme in the presence of 500 μM acetyl‐CoA and a varying concentration of **3**. Under these conditions, the activity of 5THNAT is characterized by a *k*_cat_ of 2.2 ± 0.1 s^−1^ and a *K*_M_ of 58 ± 3 (**Table** **1**). Keeping [**3**] at 1000 μM, varying concentration of acetyl‐CoA, we determined a *k*_cat_ of 1.8 ± 0.1 s^−1^ and a *K*_M_ of 73 ± 7 M. Comparison with kinetic parameters reported for other amino acid‐transforming *N*‐acetyltransferases (Table 1) suggests that **3** and acetyl‐CoA are efficient substrates for 5THNAT. Despite acetyl‐CoA being an efficient substrate, we cannot exclude the possibility that this enzyme may also accept alternative acyl‐CoAs as substrates to produce other *N*‐acyl 5‐thiohistidines, though such alternative substrates would likely be less abundant.

**Table 1:** Amino acid *N*_α_‐acetyltransferases.

enzyme	acceptor	*k*_cat_ [s^−1^]	*K*_M, Ac‐CoA_ [mM]	*K*_M,acceptor_ [mM]	lit
5THNAT	3	2.0	0.073	0.058	
Glu‐NAT	L‐Glu	0.78	0.05	5	[32]
ArgA	L‐Glu	3.3	0.15	280	[33]
D‐aa‐NAT	D‐Ser	n.a.	0.23	0.034	[34]
D‐aa‐NAT	D‐Ala	n.a.	0.23	0.054	[34]
Mpr1	azetidine‐1‐carboxlyate	33.5	0.028	1.52	[35]
Hist‐NAT	L‐His	n.a.	0.027	0.45	[36]
Asp‐NAT	L‐Asp	n.a.	0.001	0.09	[37]
Phe‐NAT	L‐Phe	n.a.	0.13	3.3	[38a–c]
NatA	phenylglycine	13.6	n.a.	0.145	[38]
BAR	phosphinothricin	23	n.a.	0.13	[22]

**Figure 5:**
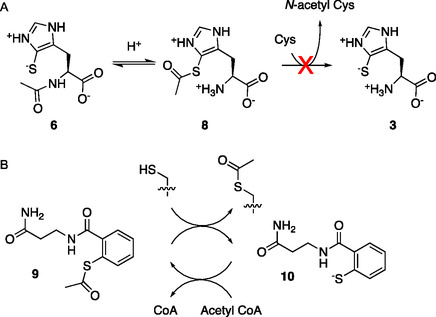
A) *N*α‐acetyl and *S*‐acetyl 5‐thiohistidine (**6** and **8**) are related by reversible *S*‐to‐*N* acetyl shift. At physiological pH, the concentration of **10** is too low to allow intermolecular acetyl transfer without catalysis. B) Catalytic acetylation of zinc‐finger proteins mediated by S‐acyl‐2‐mercaptobenzamide (**8**).^[^ ^31^ ^]^

The identification of 5THNAT raises the question about the function of **6**. Unlike *N*π‐methylation of **3** to form **1,** or *N*α‐methylation of **1** to form **4** and **5**, *N*α‐acetylation of **3** changes the overall charge of this amphiprotic metabolite from neutral to monoanionic at physiological pH. This change likely increases the affinity for metal cations, the nucleophilicity of the thiolate function, and the one‐electron reduction potential.^[11b]^ Hence, *N*α‐acetylation may optimize the performance in the same antioxidative defenses that are also served by **1**, **4,** and **5**. In addition, *N*α‐acetylation may also introduce entirely novel functions. One possibility is that **3** may act as an acetyl transfer catalyst. From the chemical synthesis of 5‐thiohistidines,^[^ ^27^ ^]^ we know that the acetyl group of **6** can migrate between the *N*α and the thiolate group via an *S*‐to‐*N*‐acyl shift (**Figure** **5**).^[^ ^28^ ^]^ Although ^1^H NMR confirms that the *N*α‐acetylated isomer **6** is dominant at neutral pH (Figure S6, Supporting Information), the *S*‐acetylated isomer **8** is stable at lower pH (pH 2, Figure S6, Supporting Information). Hence, a small fraction of **8** may be available even at physiological pH to interact with specific proteins and acetylate external *S*‐ or *N*‐nucleophiles (Figure 5). Aromatic acetyl thiolates—for example, *S*‐acetyl‐2‐mercaptobenzamide (**9,** Figure 5)—have been shown to inactivate Zinc‐finger proteins by mediating the transfer of acetyl groups from acetyl‐CoA to Zn‐coordinating Cys residues.^[^ ^29^ ^]^ To explore the ability of **6** to serve a similar function, we incubated 2 mM of purified **6** with 10 mM Cys in 100 mM phosphate buffer, pH 7.4, 100 mM NaCl, and 10 mM TCEP, at 23–26 °C for 3 days. Analysis of this reaction mixture by HPLC did not indicate the formation of *N*‐acetyl Cys, suggesting that the concentration of **8** is too low to enable acetyl transfer at a measurable rate without the assistance of a protein. The compound **6** is a stable metabolite that would need specific catalysis to serve as an acetylation reagent (Figure S7, Supporting Information).

## 3 Conclusion

In this report, we describe a novel family of *N*‐acetyl transferases (5THNAT) that produce *N*α‐acetyl 5‐thiohistidine (**6**) from 5‐thiohistidine (**3**) using acetyl‐CoA as a co‐substrate. The only other known enzyme that recognizes any 5‐thiohistidine derivative as a substrate is the ovothiol biosynthetic enzyme OvoA, which catalyzes *N*π‐methylation of **3** (**1**, Figure 1). The observation that 5THNAT does not accept **1** as a substrate suggests that *N*π‐methylation and *N*α‐acetylation are competing modifications of **3** that may serve different cellular functions. Most organisms that encode 5THNATs are gram‐negative bacteria from the phylum Bacteroidota that were isolated from cold and salty environments such as hypersaline lakes, polar oceans, and deep‐sea water (Table S1, Supporting Information), raising the possibility that **6** plays a role in cold adaptation and osmoprotection.

## Conflict of Interest

The authors declare no conflict of interest.

## Supporting information

Supplementary Material

## Data Availability

The data that support the findings of this study are available in the supplementary material of this article.
