# Supplementary material for: 5‐Thiohistidine N‐Acetyltransferase from Proteiniphilum Saccharofermentans
Source: Chembiochem. 2025 Oct 7;26(22):e202400439. doi: 10.1002/cbic.202400439 (PMC12631010; doi:10.1002/cbic.202400439)
Supplement: Supplementary file 1 — Supplementary Material [file CBIC-26-e202400439-s001.pdf]

Supporting information: "5-thiohistidine *N*-acetyltransferase from *Proteiniphilum saccharofermentans*"

Cangsong Liao,[1] David Lim,[1,2] Gladwin Suryatin Alim[1] and Florian P. Seebeck[1]*

[1] Department of Chemistry, University of Basel, Mattenstrasse 22, Basel 4002, Switzerland

[2] Molecular Systems Engineering, National Competence Center in Research (NCCR), 4058 Basel, Switzerland

Table S1: OvoA-associated *N*-acetyltransferases

Species	OvoA	Acetyl transferase	Life style
Proteiniphilum saccharofermentans	PSM36_1438	PSM36_1437	isolated from a mesophilic laboratory-scale biogas reactor
Porphyromonadaceae bacterium KH3CP3RA	SFK94343.1	SFK94319.1	-
Porphyromonadaceae bacterium	MCE5204532.1	MCE5204533.1	wastewater metagenome
Proteiniphilum acetatigenes	KUK76868.1[(a)]	KUK76869.1	terestrial metagenome
Bacteroidetes bacterium GWC1_47_7	-	A2071_00525	subsurface metagenome
Bacteroidetes bacterium GWC2_46_850	-	A2W87_06415	subsurface metagenome
Bacteroidia bacterium 43-41	-	BGO33_05835	bioreactor metagenome
Bacteroidia bacterium 44-10	OJV86344.1	OJV86345.1	bioreactor metagenome
Dysgonamonadaceae bacterium	MDD2243712.1	MDD2243711.1	landfill metagenome
Dysgonomonadaceae bacterium zrk40	QRX63579.1	QRX63578.1	-
Fermentimonas caenicola	-	ING2E5B_0833	isolated from mesophilic laboratory-scale biogas re-actors
Fermentimonas sp.	HLW09380.1	HLW09379.1	soil metagenome
Lacrimispora amygdalina	-	MDK2966352.1	isolated from sludge from a wastewater treatment plant
Bacteroidales bacterium	NLA62225.1	NLA62226.1	anaerobic digester metagenome
Bacteroidales bacterium	NLJ00282.1	NLJ00281.1	anaerobic digester metagenome
Petrimonas sp.	MDD3541560.1	MDD3541559.1	landfill metagenome
Bacteroidetes bacterium GWC1_47_7	OFX75364.1	OFX75353.1	subsurface metagenome
Paludibacter sp.	MBP6260751.1	MBP6260750.1	wastewater metagenome
Bacteroidales bacterium	NLC50160.1	NLC50159.1	anaerobic digester metagenome
Bacteroidales bacterium	NLD24846.1	NLD24845.1	anaerobic digester metagenome
Lascolabacillus sp.	MCK9500523.1	MCK9500522.1	landfill metagenome
Fermentimonas sp.	MDD4778094.1	MDD4778095.1	landfill metagenome
Paludibacteraceae bacterium	HRG04011.1	HRG04012.1	landfill metagenome
Porphyromonas macacae	NCB83629.1	WP_036851181.1	isolated from the periodontal pocket of the macaque Macaca arctoides
	MDD2298695.1	MDD2298696.1	landfill metagenome
Paludibacter sp.	MEA4850559.1	MEA4850558.1	groundwater metagenome
Paludibacter propionicigenes	ADQ80360.1	ADQ80361.1	isolated from plant residue in irrigated rice-field soil
Porphyromonadaceae bacterium CG2_30_38_12	OIP82299.1	OIP82298.1	groundwater metagenome
Paludibacter sp.	HJV78650.1	HJV78649.1	soil metagenome
Bacteroidota bacterium	NWJ52972.1[(a)]	NWJ52973.1	freshwater metagenome
Proteiniphilum sp.	MBZ4651303.1[(a)]	MBZ4651302.1	soil metagenome
Dysgonamonadaceae bacterium	HKM45318.1[(a)]	HKM45319.1	soil metagenome
Bacteroidales bacterium	MCE5332616.1	MCE5332615.1	wastewater metagenome
Prolixibacteraceae bacterium	MCF8362154.1	MCF8362155.1	lake water metagenome
Bacteroidales bacterium 36-12.	OJV37948.1	OJV37949.1	bioreactor metagenome
Bacteroidia bacterium	NCB67639.1	NCB67640.1	wastewater metagenome
Psychroflexus gondwanensis	TXE21125.1	TXE21126.1	isolated from a lake in Antarctica
Psychroflexus torquis ATCC 700755	P700755_001870	P700755_001869	Isolated from sea-ice
Psychroflexus torquis	AFU68697.1	AFU68696.1	Isolated from Antarctic sea ice
Psychroflexus planctonicus	GGE29559.1	GGE29563.1	isolated from the Lake Xiaochaidan
Psychroflexus lacisalsi	MBZ9621175.1	MBZ9621175.1	isolated from a hypersaline lake
Psychroflexus montanilacus	MBZ9652825.1	MBZ9652824.1	Antarctic sea ice
Porphyromonadaceae bacterium	NLO71005.1	NLO71004.1	anaerobic digester metagenome
Draconibacterium halophilum	QIA07859.1	QIA07858.1	Halophilic Bacterium Isolated from Marine Sediment
Psychroflexus sp. MES1-P1E	PKG43321.1	PKG43322.1	cold environments such as lakes, polar oceans, deep-sea water
Psychroflexus salis	GGE17211.1	GGE17215.1	strictly aerobic, isolated from salt lake
Bacteroidota bacterium isolate	MBP1675610.1	MBP1675609.1	wetland metagenome
Paludibacteraceae bacterium	MBN2766469.1	MBN2766468.1	freshwater metagenome
Psychroflexus longus	MBZ9779501.1	MBZ9779500.1	isolated from salt lakes
Psychroflexus curvus	MBZ9628299.1	MBZ9628298.1	isolated from salt lakes
Draconibacterium orientale	SES69931.1	SES69964.1	isolated from a marine sediment
Flavobacteriaceae bacterium	MCH8536046.1	MCH8536045.1	bioreactor metagenome
Draconibacterium orientale strain DSM 25947	SAMN05444285_101236	SAMN05444285_101237	-
Psychroflexus sp. MES1-P1E	CXF67_05685	CXF67_05690	isolated from a salt lake
Bacteroidales bacterium	BGO29_12695	BGO29_12700	bioreactor metagenome
Bacteroidales bacterium	NLY24767.1	NLY24766.1	anaerobic digester metagenome

[(a)]OvoA homologs without C-terminal methyltransferase

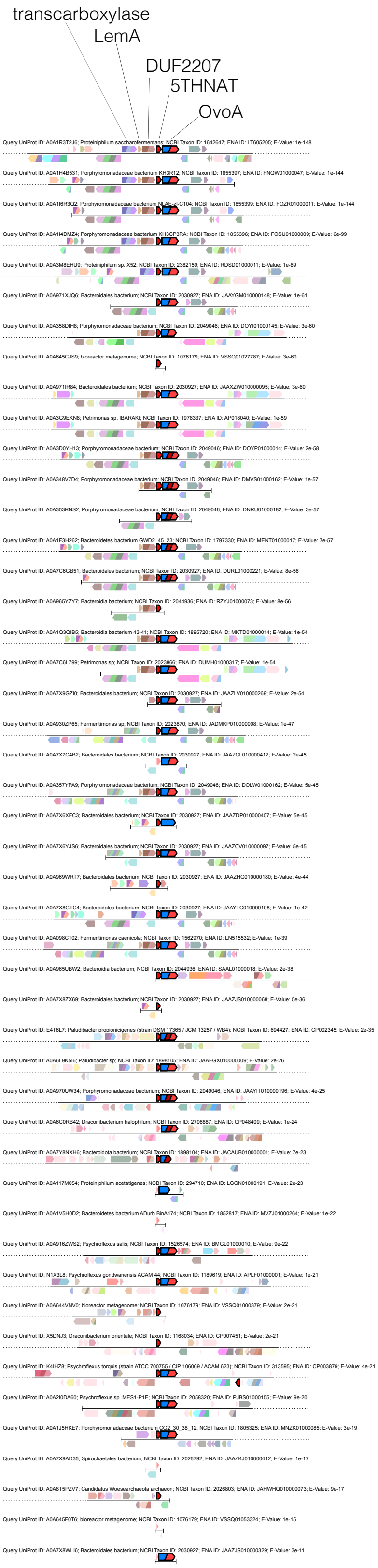

Figure S1. Genome Neighborhood analysis of 5THNAT homologs. The five-gene cluster in the genome of *Proteiniphilum saccharofermentans* coding for a transarboxylase, LemA, DUF2207, 5THNAT and OvoA is not a highly conserved motif, suggesting that the first three proteins are not involved in the production and utilization of *N*α-acetyl 5-thiohiohistidine.

Recombinant 5-thiohistidine acetyltransferase. The codon-optimized gene coding for the putative *N*-acetyltransferase (A0A1R3T2J6, Table S1) from *Proteiniphilum saccharofermentans* was purchased from Genscript. This fragment was inserted as an Nde1/Xho1 fragment into a pET28a plasmid to code for the following recombinant protein:

```
>5THNAT (A0A1R3T2J6)
MGSSHHHHHHSSGLVPAGSHMYLVSGSSLFYGWFKKNMDSIKGTVEIYDLDEYNYPKFRGQLIGLYLHAFTTGDYSQHIDRETVEVTLDDILQDGSGCMAFVG
NRLVGLLAAFPLKRDPDFPADTCRDIPVGEAIYIAEVMVHADYRGRRIASKMLNTYLQEKSENYSYAVIRVWEKNMPALELYKKLGFLPVATISQQKLSANKE
EFVMNKLYLAVKIIDN*
```

This plasmid was transformed into competent BL21(DE3)pLysS cells by heat shock. Cells were cultivated in LB medium at 37°C. When the cell culture reached OD_{600} = 0.6, gene expression was induced by the addition of 0.25 mM of IPTG. The culture was then incubated at 18 °C for 15 hours. Cells were harvested by centrifugation at 4°C and 17600 × g for 20 min. The pellets were resuspended in lysis buffer (50 mM phosphate buffer, 300 mM NaCl, pH 8.0) and lysed by microfluidizer or sonication followed by centrifugation of lysate at 4°C and 68900 × g for 60 min. The cleared supernatant was supplemented with 10 mM and Ni-NTA resin (Qiagen GmbH) and incubated at 4°C for 20 min. Subsequently, the resin was washed with 10 ml lysis buffer with added 10 mM imidazole, with 10 ml lysis buffer with added 20 mM imidazole, and eluted in lysis buffer supplemented with 250 mM imidazole. The purified protein was dialyzed into 50 mM phosphate buffer (pH 8.0) The homogeneity of the purified proteins was assessed by SDS-PAGE. Recombinant OvoA was produced as previously described.[1]

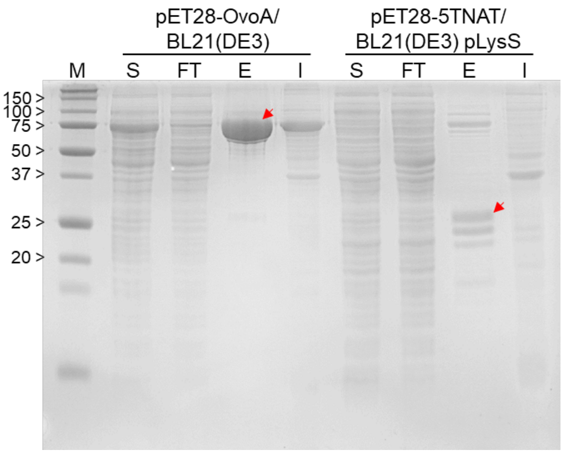

Figure S2. SDS-PAGE (12%) of recombinant OvoA and 5THNAT in *E. coli*. Cell was grown in TB media with normal and cold-shocked with ethanol condition for OvoA and 5THNAT, respectively. Protein induction was performed with 0.25 mM IPTG at 18°C for 21 h. M, marker; S, soluble; FT, flow-through; E, elute (*i.e.,* purified); I, insoluble.

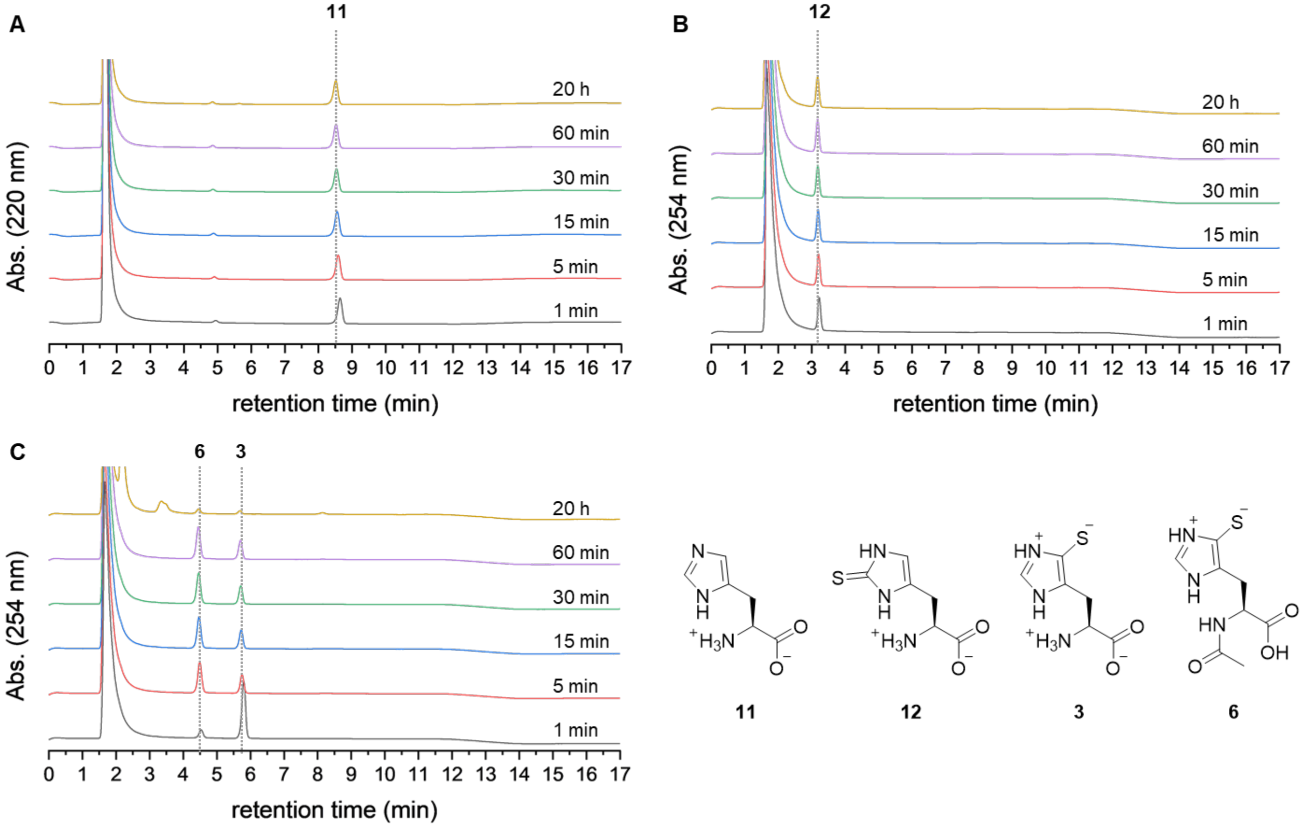

Figure S3. Ion-exchange HPLC (IE-HPLC) traces of 5THNAT reaction with 0.5 mM histidine (A), 2-thiohistidine (B), or 5-thiohistidine (C) at varying time points. Solutions containing 0.5 mM substrate, 1 mM acetyl-CoA, 2 μM 5THNAT, 2 mM TCEP, 20 mM NaCl in 50 mM Na-phosphate buffer (pH 8.0) were incubated at 25°C. Reaction aliquots were quenched by the addition of one volume equivalent of 2% trichloroacetic acid aqueous solution. Analysis of these HPLC data suggests that no detectable new product is formed with histidine or 2-thiohistidine as acetyl acceptor under conditions that acetylate **3**.

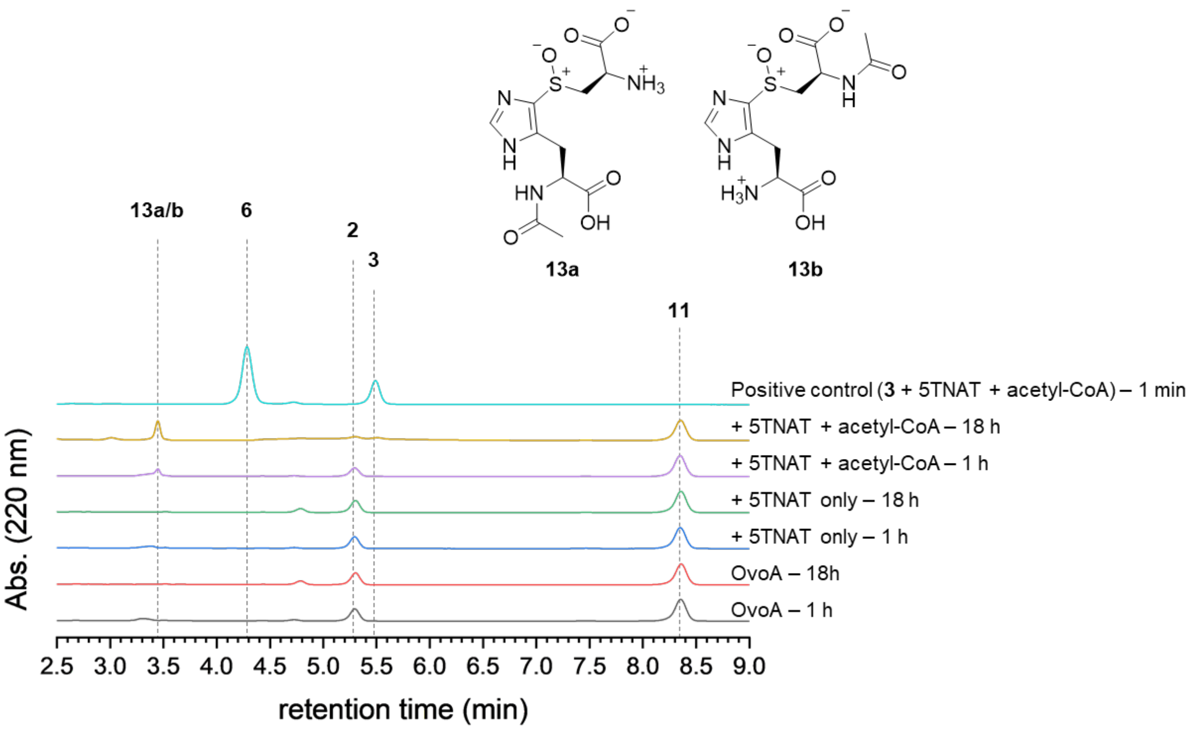

Figure S4. Ion Exchange HPLC (IE-HPLC) trace of 5THNAT reaction with sulfoxide **2** and acetyl-CoA. A solution containing **2** was generated by incubating 20 µM OvoA with 1 mM histidine, 2 mM cysteine, 20 µM $FeSO_4$, 1 mM ascorbate, 2 mM TCEP, 20 mM NaCl in 50 mM Na-phosphate buffer (pH 8.0) for 18 h at 25°C. This solution was supplemented with 10 µM 5THNAT, 1 mM acetyl-CoA, and 2 mM TCEP. Reaction aliquots were quenched by the addition of one volume equivalent 2% TCA solution. HPLC analysis (Luna 5µm SCX 100 Å 150 × 4.6 mm, Phenomenex) of these samples suggests that acetylation of sulfoxide **2** by 5THNAT proceeds at least 100-fold more slowly than acetylation of the native substrate **3** (compare with Figure S3). Recombinant OvoA was produced as described previously.[2]

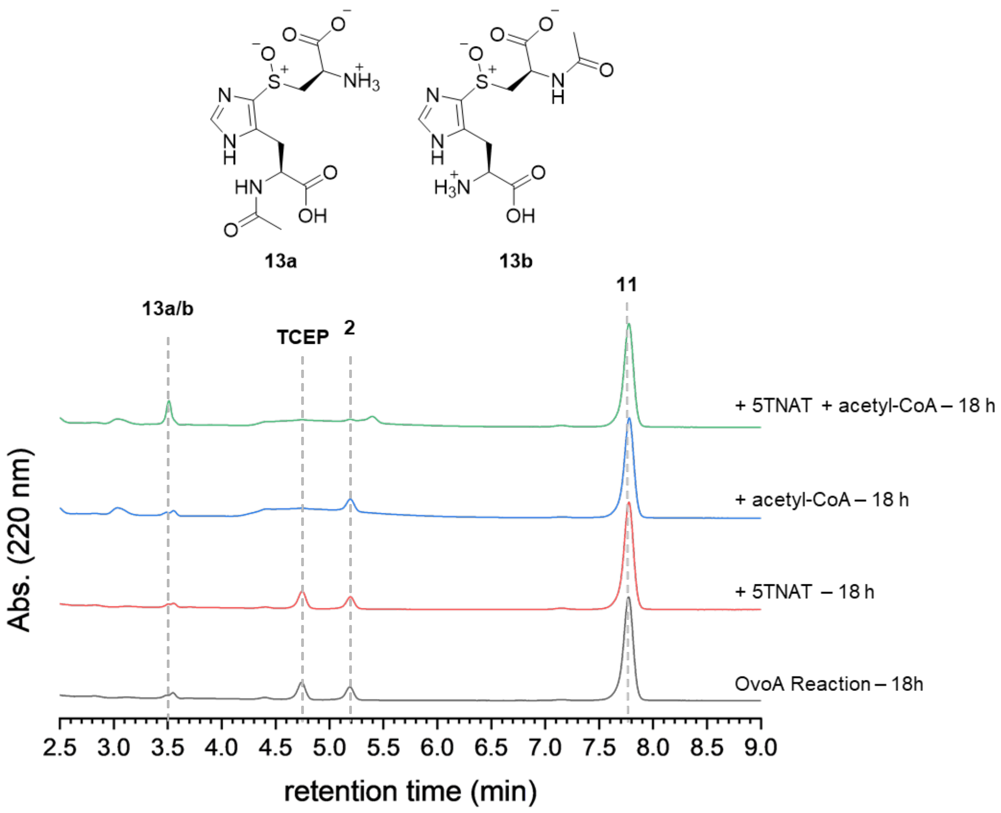

Figure S5. Ion Exchange HPLC (IE-HPLC) trace of 5THNAT reaction with sulfoxide **2** and acetyl-CoA. Same experimental conditions as described in Figure S3. This additional data shows that **2** is converted to a new product only in the presence of 5THNAT and acetyl-CoA. We have not investigated whether acetylation occurs at the histidinyl-Nα (**13a**) or at the cysteinyl Nα (**13b**)

IE-HPLC. HPLC (Nexera series, Shimadzu) was equipped with DAD (SPD-M40) with selected detection wavelength at 220 nm and 254 nm. The analysis was performed using Luna 5μm SCX 100 Å 150 × 4.6 mm (Phenomenex) column set at 40 °C, and sample injection was set to 10 μL. Binary gradient was used comprising buffer (A) 20 mM H_3PO_4 and (B) 20 mM H_3PO_4 + 1 M NaCl (adjusted to pH 2.0 with NaOH). The initial condition (5% B) was increased linearly to 20% B over 9 min. The gradient was increased to 99% over 2 min, held at 99% for 3 min, and followed by 3 min wash before the next measurement. For figure S4, the first and second gradient was set to 7 and 0.5 min, respectively.

Synthesis of *N*-acetyl-5-thiohistidine (6). L-histidine hydrochloride monohydrate (10 mg, 48 μmol) was stirred in H_2O (340 μL), conc. HCl (4 μL, 48 μmol) was added, and the reaction mixture was cooled to 0 °C. *N*-bromosuccinimide (11 mg, 62 μmol) was added to the vigorously stirring reaction mixture. After 3 mins, thioacetic acid (134 μL, 191 μmol) was added and the reaction mixture was stirred at 0 °C. After 1 h, the reaction mixture was neutralized to pH 7.5 using sat. aq. $NaHCO_3$, diluted with water (10 mL) and the aqueous phase was washed with EtOAc (2 x 10 mL), and concentrated *in vacuo* to give *N*-acetyl-5-thiohistidine **6** as a brown solid; δ_H (400 MHz, D_2O + DCl) 2.00 (3H, s, C*H*₃CONH), 2.99 (1H, dd, *J* 15.2 Hz, 4.5 Hz, β-CH), 3.15 (1H, dd, *J* 15.1 Hz, 9.0 Hz, β-CH), 8.22 (1H, s, H-2).

Synthesis of 5-Thioacetyl-L-histidine (8). L-histidine hydrochloride monohydrate (10 mg, 48 μmol) was stirred in H_2O (340 μL), conc. HCl (4 μL, 48 μmol) was added, and the reaction mixture was cooled to 0 °C. *N*-bromosuccinimide (11 mg, 62

μmol) was added to the vigorously stirring reaction mixture. After 3 mins, thioacetic acid (134 μL, 191 μmol) was added and the reaction mixture was stirred at 0 °C. After 1 h, the reaction mixture was diluted with water (10 mL) and the aqueous phase was washed with EtOAc (2 x 10 m) and concentrated *in vacuo.* Purification by cation-exchange chromatography (Amberlite IR-120 (H^+ form) and recrystallization (H_2O/EtOH) gave 5-thioacetyl-L-histidine **8** as a light brown solid; δ_H (400 MHz, D_2O + DCl) 2.00 (3H, s, C*H*3COS), 3.05 (1H, dd, *J* 15.1 Hz, 8.1 Hz, β-CH), 3.17 (1H, dd, *J* 15.0 Hz, 5.6 Hz, β-CH), 8.88 (1H, s, H-2).

Conversion of 8 to 6 via *S*-to-*N* acyl shift. L-Histidine hydrochloride monohydrate (10 mg, 48 μmol) was stirred in D_2O (340 μL), conc. DCl (4 μL, 48 μmol) was added, and the reaction mixture was cooled to 0 °C. *N*-bromosuccinimide (11 mg, 62 μmol) was added to the vigorously stirring reaction mixture. After 3 mins, thioacetic acid (134 μL, 191 μmol) was added and the reaction mixture was stirred at 0 °C. After 1 h, the reaction mixture was neutralized to pH 7.5 using solid $NaHCO_3$, and directly submitted for ^{1}H NMR analysis (Figure S3).

Procedure for the investigation of acyl transfer potential of 6. 2 mM of **6** was incubated with 10 mM L-cysteine in 100 mM phosphate buffer, pH 7.4 containing NaCl (100 mM) and TCEP (10 mM) and the reaction mixture was allowed to stir at room temperature. After 72 h, an aliquot (20 μL) was withdrawn and diluted with H_2O + 0.1 % trifluoroacetic acid (180 μL) and subjected to RP-HPLC analysis.

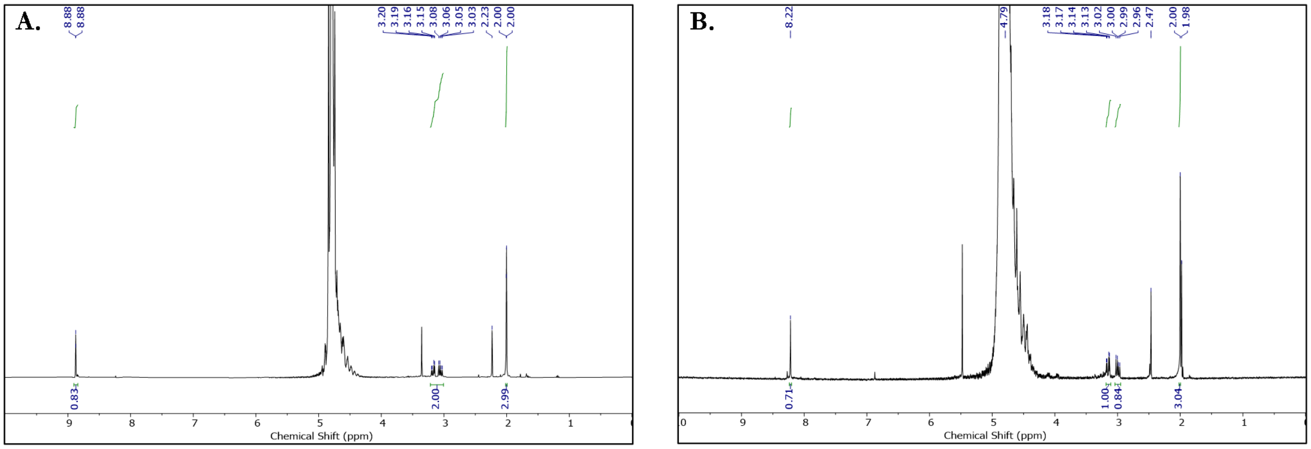

Figure S6. ^{1}H NMR spectra for. **8** (A) at pH 2 (A) and after neutralizing the solution to pH 7.5 (B). The downfield shift of H-2 from 8.88 ppm to 8.22 ppm is diagnostic for the expected *S*-to-*N* acyl shift that occurs upon neutralization.

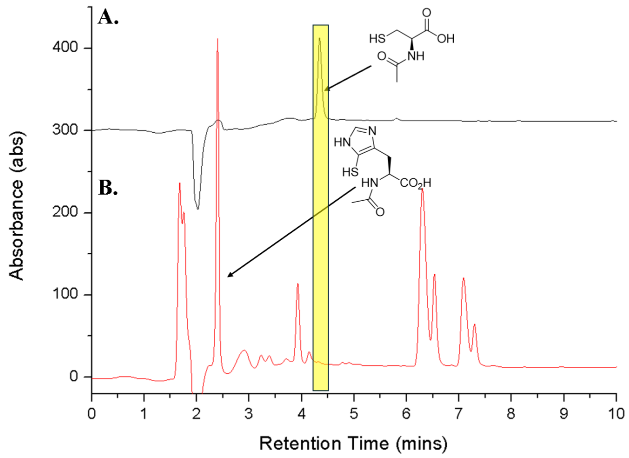

Figure S7. Investigation of the acyl transfer potential of **6**. 2 mM of synthetic **6** was incubated with 10 mM L-cysteine in 100 mM phosphate buffer, pH 7.4 containing 100 mM NaCl and 10 mM TCEP at 22 – 25°C (room temperature). After 72 h, an aliquot (20 µL) was withdrawn and diluted with H_2O + 0.1 % trifluoroacetic acid (180 µL) and subjected to RP-HPLC analysis. **A:** Reference HPLC chromatogram of *N*-acetyl-L-cysteine; **B:** Reaction mixture after 3 days.

[1] A. Braunshausen, F. P. Seebeck, *J. Am. Chem. Soc.* **2011**, *133*, 1757-1759.

[2] G. T. Mashabela, F. P. Seebeck, *Chem. Commun.* **2013**, *49*, 7714 - 7716.
